# Healing of Humerus Non-union Fracture Using Recombinant Human Bone Morphogenetic Protein With Bone Graft Compared to Bone Graft Alone: A Systematic Review and Meta-Analysis

**DOI:** 10.7759/cureus.71732

**Published:** 2024-10-17

**Authors:** Mohammed S Alhakbani, Abdulaziz A AlQahtani, Wail A AlTreef, Aljoharah I Aleisa, Haif K Al Gahtani, Mohammed N Alnasser

**Affiliations:** 1 Orthopedic Surgery, Prince Sultan Military Medical City, Riyadh, SAU; 2 College of Medicine, Imam Mohammad Ibn Saud Islamic University, Riyadh, SAU; 3 College of Medicine, Princess Nourah Bint Abdulrahman University, Riyadh, SAU

**Keywords:** allograft, autograft, bone graft, long bone non-union fracture, recombinant human bone morphogenetic protein

## Abstract

Non-union fractures of the humerus present significant challenges in orthopedic surgery, often requiring advanced treatments to achieve successful bone healing. The study aimed to compare the use of recombinant human bone morphogenetic protein (rhBMP) with bone grafts versus bone grafts alone for treating humerus non-union fractures with regard to healing rate and complications. Six databases, PubMed, ScienceDirect, The Cochrane Library, Scopus, Web of Science, and Google Scholar, were searched for relevant literature using the Preferred Reporting Items for Systematic Reviews and Meta-Analyses (PRISMA) guidelines and studies were selected according to the set eligibility criteria. Quality assessment was performed using the Mixed Methods Appraisal Tool for randomized controlled trials (RCTs) and non-RCTs. Review Manager (RevMan) version 5.4 (2020, The Cochrane Collaboration, London, United Kingdom) was utilized for meta-analysis at a significance level of 0.01. Eighteen research papers were included for qualitative and quantitative analysis. Due to the unavailability of RCTs, data from the two studies were combined. The pooled data from 16 studies for effectiveness regarding union achieved for rhBMP with bone graft versus bone graft alone was 0.65 (95%CI: 0.07-6.38, *I^2^*=67%, p=0.02). For rhBMP-2 and rhBMP-7 with bone graft, the pooled data was 0.09 (95%CI: 0.00-3.63) with high heterogeneity (*I^2^*=88%) and statistically significant differences (p<0.00001). In the sub-group analysis, the pooled data for infection rate was 1.18 (95%CI, 0.37-3.73) with 39% heterogeneity and a non-significant difference (p=0.10). Adding rhBMP to bone grafts may not significantly improve union rates compared to bone graft alone in humerus non-union fractures. However, the trend shows increased infection rates with rhBMP usage. Further high-quality RCTs are warranted to confirm these findings and elucidate the optimal management strategy for humerus non-union fractures.

## Introduction and background

Non-union bone fracture is an orthopedic condition characterized by a prolonged lack of healing, resulting in significant clinical burden and morbidity, with an estimated global prevalence of nine million cases annually [1,2]. No precise timeline exists for defining a non-union. The general guideline is three months for a delayed union and six months to declare a non-union. Non-union is defined as no healing within six months or more [3]. This condition commonly affects long bones such as the forearm, tibia, humerus, femur, and clavicle [2,4]. Non-union fractures in the humerus or long bones occur in approximately 5-10% of patients [5], with rates ranging from 45% in the tibia, 12.5% in the femur, 9% in the humerus, 7% in the ulna, and 5% in the radius [6-8]. Similarly, non-union humeral fractures are frequently seen in clinical practice, occurring in approximately 2-10% of cases after conservative management and 30% after surgical treatment [9].

Furthermore, non-unions can occur due to different biological and patient-related risk factors. Biological factors include the location of the fracture site, bone displacement, surgical treatment, fixation type, comminution, treatment delay, inadequate treatment, and wound infection [10,11]. Patient-related risk factors include age, obesity, smoking, diabetes, and non-steroidal anti-inflammatory drug (NSAID) use [10]. Furthermore, surgeons face significant challenges in managing non-unions, especially considering the mechanism of trauma, patient comorbidities, previous treatments, infection rates, and the poor quality of large bone defects [12]. Therefore, appropriate management of non-union fractures is necessary.

Management of non-union fractures often requires surgical intervention to heal and restore bone function because patients often experience pain in the upper extremity, prolonged disability, and the inability to complete tasks during work; thereby, impairing their quality of life [13] and also associated with psychological distress, loss of function, and economic well-being [2,4]. Various techniques, including fixed-angle locking plates, allografts, intramedullary nailing, external fixators, and their combinations, are available for treating non-union fractures [12]. Bone grafts remain a cornerstone for treating non-union fractures [14]. They are particularly effective for managing bone defects, delayed non-unions or unions, and spinal fusion [14]. However, bone grafting can lead to complications such as pain, scarring, infections, blood loss, and donor-site morbidity [15]. Moreover, autografting has shown limited efficacy in managing the challenges faced by a subgroup of patients with long bone fractures, accounting for 5-20% of cases with delayed or inconsistent treatment [16].

Recently, considerable interest has emerged in using recombinant human bone morphogenetic protein (rhBMP) as an adjunct to conventional bone grafts for managing non-union fractures [17]. rhBMP has strong bone induction properties and can promote fracture healing and repair other bone defects [18]. Belonging to a family that promotes bone formation and regeneration, rhBMP mimics the activity of native osteogenic proteins, enhancing bone density and promoting healing in clinical settings [19]. Additionally, rhBMPs are signaling molecules that belong to the transforming rh-β proteins superfamily. Initially, these proteins were identified to have the ability to induce bone formation [20]. More than 20 bone morphogenetic proteins (BMPs) have been identified; however, only one BMP subset can induce de novo formation of bone. Other studies have shown that BMPs can induce the differentiation of stem cells into osteogenic cells and mesenchymal stem cells, leading to bone production [21-25].

Systematic reviews and meta-analyses are usually required to synthesize existing research on a particular topic to provide a comprehensive and unbiased summary of the evidence. This approach integrates findings from multiple studies, increases statistical power, and improves the accuracy of effect estimates. Therefore, the present study may provide valuable insights into the benefits and risks of these treatments. Given the clinical complexity and complications associated with humeral non-union fractures, including limited soft tissue coverage, biomechanical considerations, and variable outcomes with conventional techniques, a growing interest exists in exploring adjuvant therapies like rhBMP. These therapies aim to improve orthopedic healing and patient outcomes, focusing on key measures such as bone fusion rates, time to union, complication rates, and functional outcomes. Therefore, the present systematic review and meta-analysis aimed to compare the use of rhBMP with bone graft versus bone graft alone in treating humerus non-union fractures.

## Review

Methods

This was a systematic review and meta-analysis, following the guidelines provided by the Preferred Reporting Items for Systematic Reviews and Meta-Analyses (PRISMA) [26].

Search Strategy and Selection Criteria

An advanced literature search was performed using six databases, namely ScienceDirect, PubMed, Scopus, Google Scholar, The Cochrane Library, and Web of Science, between August 2005 and March 2024. Different keywords such as recombinant human bone morphogenetic protein, rhBMP, bone morphogenetic proteins, BMPs, bone graft, bone transplantation, and humerus ununited, were used. Boolean operators (OR and AND) were used to combine keywords (see Appendix A).

Two independent reviewers evaluated the search strategy according to the three-phase selection process: (i) In the first phase, different databases were searched for the identification of relevant literature, (ii) in the screening phase, studies were screened according to the aim of the study through title and abstracts, and (iii) in the eligibility phase, studies were finalized according to the inclusion/exclusion criteria via full-text assessment and the PRISMA guidelines for transparency and reproduction of the selection process. Any disagreement was resolved among the reviewers through consultation with a third reviewer, and the issue was resolved according to the eligibility criteria set for the inclusion of studies.

Inclusion and Exclusion Criteria

Inclusion criteria: Studies were included based on different inclusion criteria. (i) All randomized controlled trials (RCTs), clinical trials, prospective studies, and retrospective studies included a rhBMP sample with bone graft used in humerus non-union compared with the control or comparator group. (ii) Studies included a sample of bone grafts alone with no additives used in the humerus for all types of non-union. (iii) Participants were aged 18 years or older in both rhBMP with bone graft and bone graft alone groups because the skeletal maturity and bone healing processes differ significantly between adults and younger individuals. In adults, bone growth has typically ceased, and the regenerative capacity is generally lower compared to children or adolescents. This age group allows for a more accurate assessment of the efficacy and safety of rhBMP in enhancing bone healing and regeneration in a population where natural bone healing might be less robust. (iv) Studies reported the time to achieve union and the rate of union in both groups. (v) All studies were conducted from and after August 2005 and (vi) were published in English with fully accessible texts.

Exclusion criteria: (i) Studies with no humerus sample for both groups. (ii) Studies that did not specify reports for the humerus non-union sample. (iii) Non-comparative studies or those not reporting outcomes or failures by subgroups. (iv) Non-human or preclinical studies. (v) Studies with incomplete or un-extractable data. (vi) Studies that did not report time to achieve union and rate of union in both groups. (vii) Studies with additional additives in both groups (i.e., demineralized bone matrix (DBM), platelet-rich plasma (PRP)). (viii) Cross-sectional studies, case series, reviews, and case reports. (ix) Studies conducted before August 2005 and (x) published in other than English language.

Data Extraction

Two independent reviewers extracted the data using pre-defined variables in Microsoft Excel (Microsoft Corporation, Redmond, Washington, United States). The included variables were study characteristics (authors, country, study design, and sample size), participant characteristics (age, sex, fracture type, medical history, and any operation performed), intervention characteristics (BMP, bone graft type, and surgical fixation type), and outcomes (blood loss in the operating room, infection rate, time to radiological union, clinical outcomes, and range of motion (ROM)).

Quality Assessment

For methodological quality assessment of non-RCTs, the Mixed Methods Appraisal Tool (MMAT) was used to rate the quality based on the degree to which each study’s methodology met predetermined criteria (see Appendix B) [27]. Studies were classified as either “low (score ≤3)” or “high (score >3)” depending on the reviewer’s response: “yes” (1 point) or “no” (0 points) [28].

Statistical Analysis

Microsoft Excel was used to construct tables for qualitative data. For quantitative data, a meta-analysis was performed to compare union achieved, BMP-2 and BMP-7 for the healing of non-union fracture and pre and post-infection rate by applying a random-effects model using Review Manager (RevMan) version 5.4 (2020, The Cochrane Collaboration, London, United Kingdom). Odds ratios (ORs) were calculated for dichotomous variables, with 95% confidence intervals (CIs) provided for all effect sizes. Statistical heterogeneity among the studies was computed using Cochrane’s Q test and I2, with >75% indicating high heterogeneity, >25-75% indicating medium heterogeneity, and <25% indicating low heterogeneity. Outcomes were reported as forest plots. Furthermore, the chi-square test was used to assess differences, and the statistical significance was set at p<0.01.

Results

Literature Search

**Figure 1:**
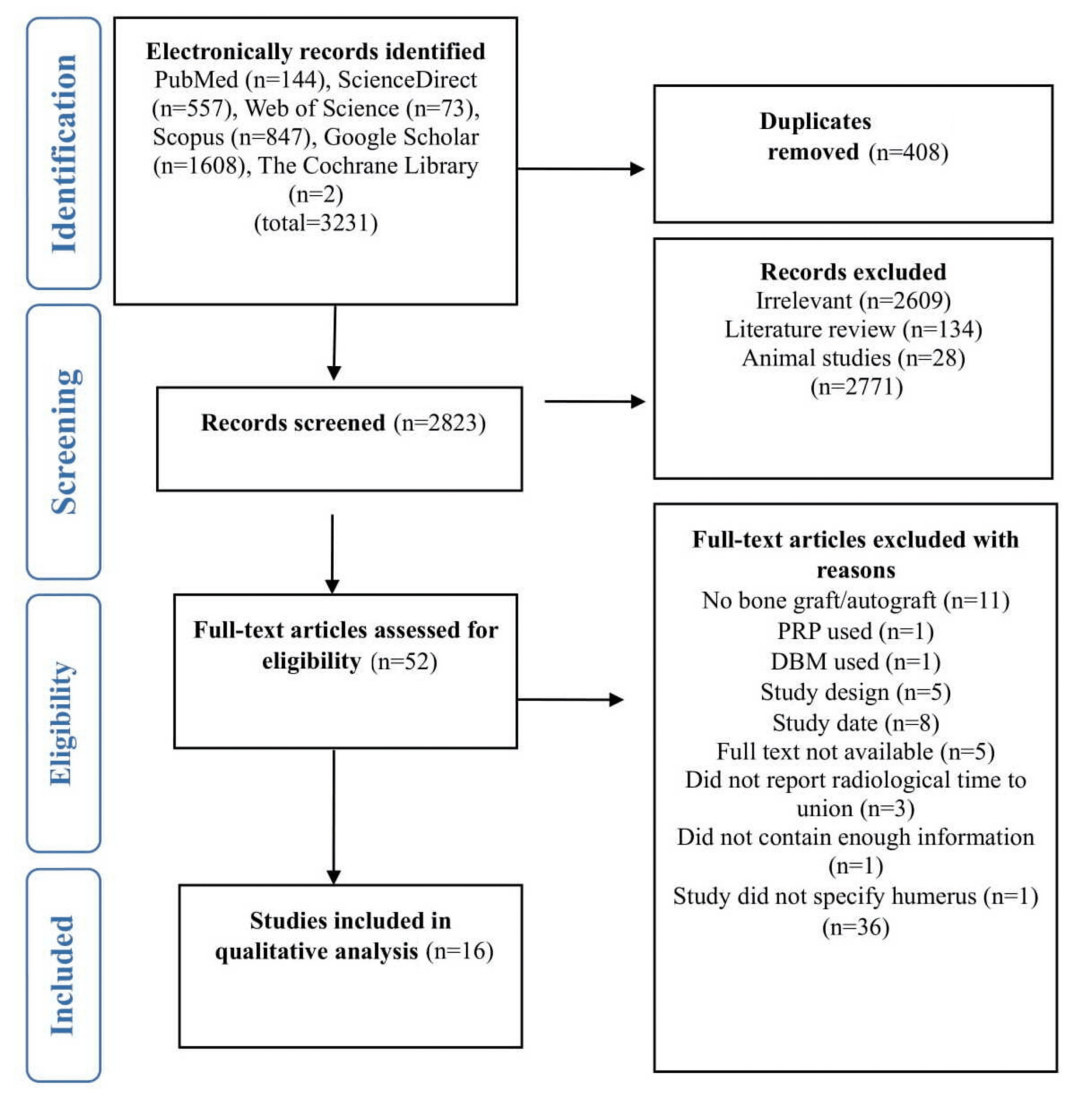
PRISMA flow chart for the selection of the studies PRISMA: Preferred Reporting Items for Systematic Reviews and Meta-Analyses

Initially, 3,231 research articles were retrieved from different databases, including PubMed, ScienceDirect, The Cochrane Library, Scopus, Web of Science, and Google Scholar. During the identification phase, 408 duplicate articles were removed. From the 2,823 remaining articles, 2,771 were excluded based on title and abstract screening for not meeting study criteria. Of the 52 articles eligible for full-text assessment, 16 were deemed suitable for qualitative and quantitative analyses. The remaining research articles were excluded for different reasons (Figure 1).

General Characteristics

rhBMP with bone graft: Most studies were conducted in the United States (n=2) and Germany (n=2), with one study each from the United Kingdom, Italy, Greece, Austria, India, and Pakistan (Table 1). Prospective study designs were most common, followed by retrospective [29] and RCT [30]. rhBMP with bone graft treatment involved 109 patients with non-union humerus fractures, ranging from three to 23 patients [31,32]. Among the 109 patients, 47 were male and 59 were female, with the number of male patients ranging from one [31] to nine [32] and female patients from two [33] to 14 [32]. Patients were over 40 years of age, with mean ages ranging from 43.3 years [31] to 56.6 years [32].

**Table 1:** General characteristics of participants treated with rhBMP and bone graft and bone graft alone NA: not available; rhBMP: recombinant human bone morphogenetic protein; RCT: randomized controlled trial

Study	Country	Study design	Sample size, n	Male, n	Female, n	Age (years), mean
rhBMP with bone graft						
Bong et al. [32]	United States	Prospective	23	9	14	56.6
Dimitriou et al. [31]	United Kingdom	Prospective	3	1	2	43.3
Calori et al. [30]	Italy	RCT	15	8	7	44
Moghadam [34]	Germany	Prospective	3	NA	NA	45
Takemoto et al. [35]	United States	Prospective comparative	16	9	7	47.4
Papanagiotou et al. [36]	Greece	Prospective	10	3	7	46
Singh et al. [37]	India	Prospective	14	3	11	51.07
Hackl et al. [29]	Austria	Retrospective comparative	13	7	6	49
Fuchs et al. [38]	Germany	Prospective	6	3	3	55.33
Janjua et al. [33]	Pakistan	Prospective	6	4	2	50
Total patients			109	47	59	
Bone graft alone						
Hsu et al. [39]	Taiwan	Retrospective	105	66	39	46.2
Li et al. [40]	China	Retrospective	12	8	4	34.75
Marinelli et al. [41]	Italy	Retrospective	57	31	26	49
Rollo et al. [42]	Italy	Prospective	16	7	8	62.3
Pollon et al. [43]	France	Retrospective	16	6	10	52
Feng et al. [44]	China	Retrospective	15	9	6	45.3
Takemoto et al. [35]	United States	Prospective comparative	9	6	3	43.4
Hackl et al. [29]	Austria	Retrospective comparative	9	6	3	51
Fuchs et al. [38]	Germany	Prospective	6	3	3	48.33
Total patients			245	142	102	

Bone graft alone: Similarly, studies utilizing bone graft alone were reported from China (n=2), Italy (n=2), and single studies from the United States, Taiwan, France, Austria, and Germany (Table 1). Most studies followed a retrospective design [29,34-41], with some following a prospective design [35,38,42]. Furthermore, 245 patients with non-union humerus fractures were treated with bone grafts alone, with a minimum of six patients [38] and a maximum of 105 patients [39]. Among the 245 patients, 102 were male, ranging from three [38] to 66 [39], and 99 were female, ranging from three to 39 [40,41]. Patients were over 30 years of age, with mean ages ranging from 34.75 years [40] to 62.3 years [42].

Intervention Characteristics

The results of the comparison between using rhBMP with bone graft versus bone graft alone in treating humerus non-union fractures are summarized in Appendix C. In the rhBMP group, the number of participants using nicotine ranged from 0 to 8. Some studies reported open fractures; however, most fractures were closed, with closed fracture cases ranging from 2 to 21 [31]. Types of non-union included atrophic, septic, oligotrophic, and hypertrophic. The mean number of operations before rhBMP with bone graft administration ranged from 0.83 to 1.8, with varying proportions of rhBMP-2 and rhBMP-7 usage. Autograft and allograft usage varied across studies, with initial treatments including conservative management, open reduction internal fixation, external fixation, and intramedullary nail fixation. Three patients underwent reoperation in a single study [35]. In the bone graft alone group, studies reported varying distributions of open and closed fractures, along with atrophic, oligotrophic, and hypertrophic non-union. The mean number of operations before bone grafting ranged from 1 to 2.25 [38,44], with different initial treatment strategies and reoperation rates. These findings provide insights into the characteristics and treatment patterns of humerus non-union fractures across different study populations and interventions.

Clinical Outcomes of Interventions

In the rhBMP with bone graft treatment group, four was the maximum number [36], and one was the minimum number [32] of patients who had preoperative infection. Postoperatively, the infection rate was reduced to one [29]. The minimum and maximum duration for radiological union were 106.5 and 714.5 days, respectively [30,37]. Most patients achieved union, except in a few studies where 100% union was not achieved [36,38]. The shortest follow-up period was nine months [34], and the longest was 67.2 months [36]. The maximum duration from injury to operation was 31.4 months [37].

Similarly, in the bone graft alone treatment group, the maximum number of patients with pre- and postoperative infections was three and seven, respectively [43]. The shortest time for radiological union was 98 days [41], and the longest was 410 days [30]. After the intervention, the longest follow-up period was 78 months, and the longest duration from injury to operation was 126 months (Table 2) [43,44].

**Table 2:** Clinical outcomes of rhBMP with bone graft and bone graft alone treatment of patients NA: not available; rhBMP: recombinant human bone morphogenetic protein

Study	Infection (preoperative), n	Infection (postoperative), n	Time to radiological union (days), mean	Achieved union, n	Follow-up time (months), mean	Duration from injury to surgery (months), mean
rhBMP with bone graft						
Bong et al. [32]	1	1	144.3	23	NA	9
Dimitriou et al. [31]	NA	NA	170.4	3	15.3	19.5
Calori et al. [30]	NA	NA	106.5	15	12.43	20.2
Moghadam [34]	0	0	146	NA	9	NA
Takemoto et al. [35]	3	0	200.7	16	12	NA
Papanagiotou et al. [36]	4	NA	139.9	9	67.2	17
Singh et al. [37]	2	NA	714.5	14	24	31.4
Hackl et al. [29]	NA	1	349.7	11	12	14.8
Fuchs et al. [38]	0	NA	273.75	5	24	NA
Janjua et al. [33]	0	NA	86.03	6	NA	NA
Bone graft alone						
Hsu et al. [39]	0	3	112	105	20	8
Li et al. [40]	0	0	176.41	12	21	10.5
Marinelli et al. [41]	0	3	98	53	48	18
Rollo et al. [42]	0	0	126.4	16	32	NA
Pollon et al. [43]	3	7	243.33	12	78	14.9
Feng et al. [44]	0	1	194.66	15	23	126.8
Takemoto et al. [35]	0	2	164.25	9	12	NA
Hackl et al. [29]	NA	1	410.62	8	12	13.5
Fuchs et al. [38]	0	NA	273.75	3	24	NA

Meta-analysis

Union Achieved

**Figure 2:**
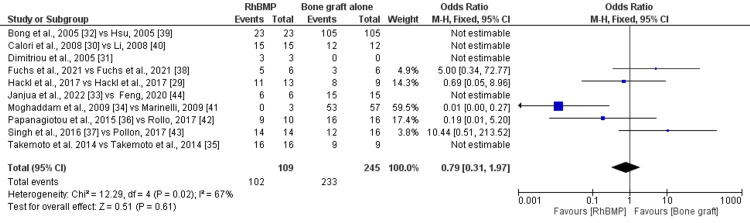
Forest plot for union achieved after the interventions rhBMP: recombinant human bone morphogenetic protein NOTE: In the image, "rhBMP" indicates rhBMP with bone graft

The pooled data of 16 studies for the effectiveness regarding union achieved for rhBMP with bone graft vs. bone graft alone was 0.65 (95%CI: 0.07-6.38) (Figure 2). The studies exhibited medium heterogeneity (I^2^=67%) and statistically non-significant differences (p=0.02).

Comparison Between BMP-2 and BMP-7

**Figure 3:**
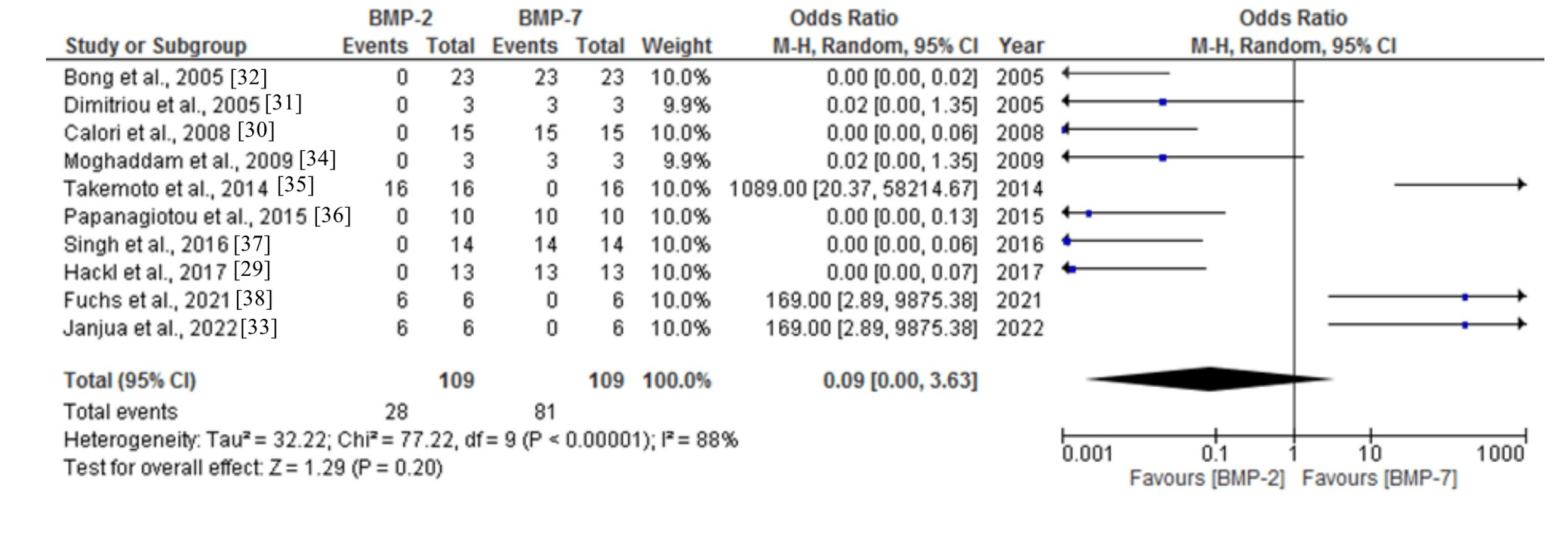
Forest plot for the use of BMP-2 and BMP-7 for the healing of non-union fracture BMP: bone morphogenetic protein

The pooled data for the utilization of BMP-2 and BMP-7 for the healing of non-union fractures was 0.09 (95%CI: 0.00-3.63) (Figure 3). The studies showed high heterogeneity (I^2^=88%) and statistically significant differences (p<0.00001).

Sub-Group Analysis for Preoperative and Postoperative Infection Rate

**Figure 4:**
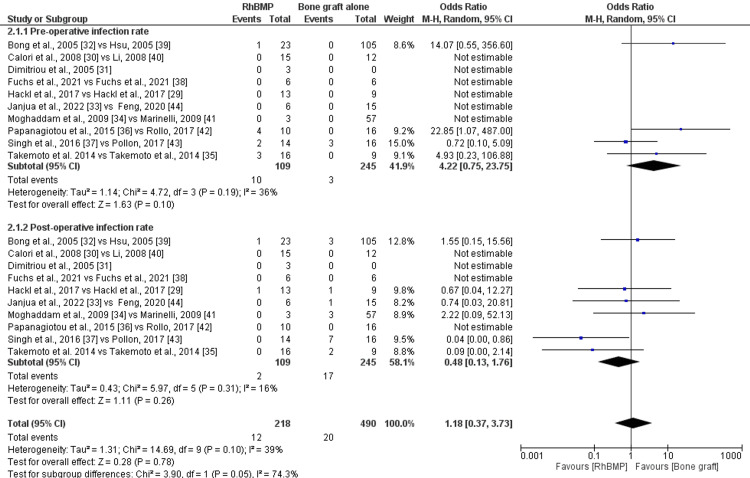
Forest plot for sub-group analysis for preoperative and postoperative rate for infections rhBMP: recombinant human bone morphogenetic protein NOTE: In the image, "rhBMP" indicates rhBMP with bone graft

The subgroup analysis compared preoperative and postoperative infection rates in the rhBMP with bone graft and bone graft alone groups. The pooled data for the preoperative infection rate was 4.22 (95%CI: 0.75-23.75) (Figure 4). The studies showed medium heterogeneity (I^2^=36%) with statistically non-significant differences (p=0.10). Similarly, for the postoperative infection rate, the pooled data was 0.48 (95%CI: 0.13-1.76) (Figure 4). The studies showed low heterogeneity (I^2^=16%) with statistically non-significant differences (p=0.31). Overall, the pooled data for infection rate was 1.18 (95%CI: 0.37-3.73), with 39% heterogeneity and a statistically non-significant difference (p=0.10) (Figure 4).

Quality Assessment

Study quality was assessed using the MMAT. All retrospective studies were of good quality according to the items presented in Appendix B, with quality scores above 3. All nine (100%) retrospective and prospective studies regarding rhBMP met four out of five MMAT criteria and showed good quality. Moreover, one RCT also achieved good quality as it met all five MMAT criteria (Table 3). Likewise, two (22.22%) studies met all five criteria and the remaining seven (77.77%) studies met four out of five criteria and were considered good quality studies. However, a major concern was identified in the sample representation domain (3.1) for all studies except two [39,41].

**Table 3:** MMAT quality assessment for included studies rhBMP: recombinant human bone morphogenetic protein; RCT: randomized controlled trial; MMAT: mixed methods appraisal tool

Study	Study design	Item					Score
rhBMP and bone graft		2.1	2.2	2.3	2.4	2.5	
Calori et al. [30]	RCT	Yes	Yes	Yes	Yes	Yes	5
		3.1	3.2	3.3	3.4	3.5	
Bong et al. [32]	Prospective	No	Yes	Yes	Yes	Yes	4
Dimitriou et al. [31]	Prospective	No	Yes	Yes	Yes	Yes	4
Moghadam [34]	Prospective	No	Yes	Yes	Yes	Yes	4
Takemoto et al. [35]	Prospective comparative	No	Yes	Yes	Yes	Yes	4
Papanagiotou et al. [36]	Prospective	No	Yes	Yes	Yes	Yes	4
Singh et al. [37]	Prospective	No	Yes	Yes	Yes	Yes	4
Hackl et al. [29]	Retrospective comparative	No	Yes	Yes	Yes	Yes	4
Fuchs et al. [38]	Prospective	No	Yes	Yes	Yes	Yes	4
Janjua et al. [33]	Prospective	No	Yes	Yes	Yes	Yes	4
Bone graft alone							
Hsu et al. [39]	Retrospective	Yes	Yes	Yes	Yes	Yes	5
Li et al. [40]	Retrospective	No	Yes	Yes	Yes	Yes	4
Marinelli et al. [41]	Retrospective	Yes	Yes	Yes	Yes	Yes	5
Rollo et al. [42]	Prospective	No	Yes	Yes	Yes	Yes	4
Pollon et al. [43]	Retrospective	No	Yes	Yes	Yes	Yes	4
Feng et al. [44]	Retrospective	No	Yes	Yes	Yes	Yes	4
Takemoto et al. [35]	Prospective comparative	No	Yes	Yes	Yes	Yes	4
Hackl et al. [29]	Retrospective comparative	No	Yes	Yes	Yes	Yes	4
Fuchs et al. [38]	Prospective	No	Yes	Yes	Yes	Yes	4

Discussion

Humerus non-union fractures pose significant challenges in orthopedic practice, often requiring interventions to stimulate bone healing and restore function [12]. Among the various treatment modalities, rhBMP combined with bone grafting has emerged as a promising approach [45]. However, the efficacy and safety of this combination therapy compared to the conventional use of bone graft alone remain debatable within the medical community. This systematic review and meta-analysis aimed to comprehensively evaluate the outcomes associated with rhBMP usage alongside bone grafting in humerus non-union fractures, offering valuable insights into its clinical effectiveness, complications, and overall impact on patient outcomes.

In this study, a non-significant (p>0.01) difference was observed between the rhBMP with bone graft and the bone graft alone groups. This may be attributed to different factors, such as heterogeneity in patient populations, including variations in fracture severity, comorbidities, and demographic characteristics, which might have influenced treatment outcomes. Moreover, variations in surgical techniques, such as the method of bone grafting and rhBMP application, could contribute to inconsistencies in results. Additionally, the sample size of the study might not have been sufficiently large to detect subtle differences between the treatment groups. Furthermore, the timing of the outcome assessments and the duration of follow-up might not have adequately captured the long-term effects of the interventions. Notably, the data was collected from different studies to compare the two groups. Another cohort of autografts had a significantly shorter union time (198 days) than combined autograft/allograft (389 days) or rhBMP-2 (217 days) [46]. Similarly, 112 non-union fractures were treated with bone graft and internal fixation, and for additional benefits for a quick and stimulating healing process, rhBMP-7 was also administered, and a non-significant (p=0.67) difference was observed among the groups. Moreover, the outcomes suggest that healing of non-union fractures may be associated with accurate surgical concepts by utilizing radical removal of non-union tissue, axis restoration, stable fixation, torsion, and length rather than the addition of rhBMP [29]. Likewise, another meta-analysis revealed a non-significant difference (p=0.74) in postoperative healing rate (risk ratio (RR): 1.01; 95%CI: 0.95-0.56) among the bone graft and bone graft with rhBMP groups [17]. Another meta-analysis with 14 studies showed a non-significant (p=0.36) difference between the bone graft and rhBMP with bone graft group regarding the healing rate of non-union (RR: 1.04; 95%CI: 0.96-1.12); however, subgroup analysis showed that the rhBMP with bone graft group had higher and better healing rates (RR: 1.35; 95%CI: 1.17-1.56) [47]. Similarly, another systematic review suggested that the addition of rhBMP is safe, has potential in the management of non-union fractures, improves union rates, reduces healing time, and ultimately enhances functional outcomes [48]. From comparing the study findings with previous literature, it can be inferred that while rhBMP combined with bone grafting shows potential in improving healing rates and reducing union times, the evidence remains inconclusive, as many studies reported non-significant differences between rhBMP with bone graft and bone graft alone groups. Despite some subgroup analyses suggesting better healing rates with rhBMP, the overall consensus across multiple studies indicates that adding rhBMP may not consistently lead to significant improvements in fracture healing compared to bone grafting alone. Therefore, while rhBMP remains a promising adjunct, its routine use should be carefully considered, with further research needed to identify specific scenarios where it offers clear clinical advantages.

Meanwhile, rhBMP-2 and rhBMP-7 added to bone grafts were both effective, a significant difference was observed favoring rhBMP-2 in our meta-analysis. Our findings align with the outcomes of another systematic scoping review, which concluded that rhBMP-2 with bone grafts is effective for non-union fractures, although no conclusive evidence was found for rhBMP-7 with bone graft [49]. Similarly, the healing rate in the rhBMP-2 group was 93% compared to 70% in the rhBMP-7 group. Additionally, the rhBMP -2 group achieved radiographic healing more quickly and bore more weight than the rhBMP-7 group [50]. A question arises: if both belong to the same rhBMP family, why does a difference in efficacy occur? The significant difference favoring rhBMP-2 over rhBMP-7 can be attributed to several factors. Firstly, rhBMP-2 and rhBMP-7 are distinct members of the BMP family, each with unique structural and functional characteristics that may influence their efficacy in promoting bone healing [51]. Secondly, differences in their mechanisms of action, such as receptor specificity and downstream signaling pathways, could contribute to variations in their effectiveness [52-54]. Additionally, variations in dosing regimens, delivery methods, types of bone grafts, and patient populations across the included studies may have influenced the comparative outcomes. The specific types of fractures (non-union) or patient characteristics within the analyzed studies may have favored the osteogenic properties of rhBMP-2 over rhBMP-7, leading to the observed significant difference in favor of rhBMP-2. Meanwhile, rhBMP-2 is also very effective in treating tibial non-union, with a 91% consolidation rate compared to 58% for rhBMP-7 [55]. However, rhBMP-7 was found to be more effective than PRP, with a 6.3% failure rate reported in the rhBMP-7 group versus 38.5% in the PRP group [56]. Furthermore, in the present meta-analysis, a non-significant (p=0.10) difference was observed in the infection rate between the rhBMP with bone graft and bone graft alone groups. However, the auto-bone graft group showed a significantly lower postoperative infection rate (12.4%) than the allograft and rhBMP groups [46]. Similarly, a massive local inflammatory reaction has been observed after the application of rhBMP for the treatment of a symptomatic forearm non-union [57]. Overall, our findings align with previous literature, reinforcing the superior efficacy of rhBMP-2 while highlighting the nuanced role of rhBMP-7 in bone healing.

Clinical Implications

The findings of this systematic review and meta-analysis suggest that rhBMP, particularly rhBMP-2, significantly enhances the healing rate and accelerates radiographic union compared to bone graft alone. This may speed recovery time, reduce the need for further surgery, and potentially improve functional outcomes for patients. Clinicians considering the therapeutic use of rhBMP should weigh these benefits against the risk of complications such as infection, local inflammatory reactions, etc. This evidence supports the widespread use of rhBMP, particularly rhBMP-2, in the humeral management of non-union fractures and reduces the burden of prolonged non-union cases.

Strengths and Limitations

A key strength of this study was the comprehensive synthesis of available evidence, providing a robust overview of the comparative effectiveness of these treatment modalities. Additionally, the inclusion of multiple studies increased the statistical power and generalizability of the findings. However, this study had some limitations. Potential heterogeneity across the included studies regarding patient characteristics, surgical techniques, and outcome measures may have introduced variability in the results. Notably, the limited number of available studies directly comparing rhBMP with bone graft and bone graft alone necessitated combining data from different studies, which could introduce bias. As stated above, rhBMP with bone grafting was remarkably effective in treating non-union in multiple bone locations, accelerating the healing process [38,55]. Therefore, future research should focus on large-scale multicenter RCTs with standardized protocols to provide robust evidence on the comparative effectiveness and safety of rhBMP with bone graft versus bone graft alone in humerus non-union fractures. This will potentially provide a clearer understanding of the benefits and limitations of adding rhBMP to bone grafts in humerus non-union treatment.

## Conclusions

This study provided valuable insights into the outcomes of utilizing rhBMP alongside bone grafting in humerus non-union fractures compared to bone grafting alone. Although the evidence suggests that adding rhBMP may not significantly improve union rates or alter infection rates, the overall efficacy and safety profile of this combination therapy remains inconclusive due to the heterogeneous nature of the included studies, the lack of a standardized protocol, and limitations such as small sample sizes. However, when rhBMP-2 and rhBMP-7 were compared, a significant difference was observed, with the meta-analysis favoring rhBMP-2. Well-designed RCTs with larger cohorts are warranted to comprehensively evaluate the comparative effectiveness of rhBMP with bone grafting in humerus non-union fractures. Such studies are crucial for aiding clinicians in making informed treatment decisions, optimizing patient outcomes, improving the quality of the management, and accelerating the healing process in humerus non-union patients.

## Appendices

Appendix A

**Table 4:** Literature search from different databases

Databases	Search terms
PubMed	((((Recombinant human bone morphogenetic protein) OR (rhBMP)) OR (Bone morphogenetic proteins)) OR (BMPs)) AND (((Humerus nonunion) OR (Humerus ununited)) OR (Upper limb nonunion)) (((((Recombinant human bone morphogenetic protein) OR (rhBMP)) OR (Bone morphogenetic proteins)) OR (BMPs)) AND ((Bone graft) AND (Bone transplantation))) AND (((Humerus nonunion) OR (Humerus ununited)) OR (Upper limb nonunion)) ("Bone grafts"[Title/Abstract] OR "Bone transplantation"[MeSH Terms] OR "Bone fixation"[Title/Abstract] OR "Surgical fixation"[Title/Abstract] OR "Allograft bone graft"[Title/Abstract] OR "Allo bone graft"[Title/Abstract] OR "Autologous bone graft"[Title/Abstract] OR "Auto bone graft"[Title/Abstract]) AND ("Humerus nonunion"[Title/Abstract] OR "Humeral non-union"[Title/Abstract] OR "Humeral shaft fracture"[Title/Abstract] OR "Humeral fracture"[Title/Abstract] OR "Humeral shaft nonunion"[Title/Abstract])
The Cochrane Library	(Recombinant human bone morphogenetic protein OR rhBMP OR Bone morphogenetic proteins OR BMPs) AND (Bone graft OR Bone transplantation) AND (Humerus nonunion OR Humerus ununited OR Upper limb nonunion)
Google Scholar	(Recombinant human bone morphogenetic protein OR rhBMP OR Bone morphogenetic proteins OR BMPs) AND (Bone graft OR Bone transplantation) AND (Humerus nonunion OR Humerus ununited OR Upper limb nonunion) (“Bone grafts” OR “Bone transplantation” OR “Bone fixation” OR “Allograft bone graft” OR “Allo bone graft” OR “Autologous bone graft” OR “Auto bone graft”) AND (“Humerus nonunion” OR “Humeral nonunion” OR “Humeral shaft fracture” OR “Humeral shaft nonunion”)
ScienceDirect	(“Bone grafts” OR “Bone transplantation” OR “Bone fixation” OR “Allograft bone graft” OR “Auto bone graft”) AND (“Humerus nonunion” OR “Humeral nonunion” OR “Humeral shaft fracture” OR “Humeral shaft nonunion”)
Web of Science	(Recombinant human bone morphogenetic protein OR rhBMP OR Bone morphogenetic proteins OR BMPs) AND (Humerus nonunion OR Humerus ununited OR Upper limb nonunion) (“Bone grafts” OR “Bone transplantation” OR “Bone fixation” OR “Surgical fixation” OR “Allograft bone graft” OR “Allo bone graft” OR “Autologous bone graft” OR “Auto bone graft”) AND (“Humerus nonunion” OR “Humeral nonunion” OR “Humeral shaft fracture” OR “Humeral fracture” OR “Humeral shaft nonunion”)

Appendix B

**Table 5:** MMAT quality assessment items MMAT: Mixed Methods Appraisal Tool

No.	Question
2.1	Is randomization appropriately performed?
2.2	Are the groups comparable at baseline?
2.3	Are there complete outcome data?
2.4	Are outcome assessors blinded to the intervention provided?
2.5	Did the participants adhere to the assigned intervention?
3.1	Are the participants representative of the target population?
3.2	Are measurements appropriate regarding both the outcome and intervention (or exposure)?
3.3	Are there complete outcome data?
3.4	Are the confounders accounted for in the design and analysis?
3.5	During the study period, is the intervention administered (or exposure occurred) as intended?

Appendix C

**Table 6:** Intervention characteristics NA: not available; BMP: bone morphogenetic protein

Study	Nicotine use (number)	Open fracture	Closed fracture	Atrophic non-union	Septic non-union	Oligotrophic non-union	Hypertrophic non-union	Number of operations before BMP (mean)	BMP-2	BMP-7	Auto-graft	Allo-graft	Combined allo and Auto	No graft	Conservative (initial)	Open reduction internal fixation (initial)	External fixation (initial)	Intra medullary nail (initial)	Open reduction internal fixation	External fixation	Intra medullary nail	Reoperation (number of persons)
rhBMP																						
Bong et al. [32]	8	2	21	23	0	0	0	1.3	0	23	4	1	18	0	NA	NA	NA	NA	21	0	2	NA
Dimitriou et al. [31]	NA	1	2	3	0	0	0	1	0	3	1	NA	1	0	2	1	NA	0	3	0	0	NA
Calori et al. [30]	5	1	14	15	0	0	0	1.8	0	15	NA	NA	NA	6	9	3	1	2	8	3	4	NA
Moghadam [34]	NA	0	3	3	0	0	0	NA	0	3	NA	NA	NA	0	NA	NA	NA	NA	NA	NA	NA	NA
Takemoto et al. [35]	3	5	11	12	0	0	4	1.4	16	0	16	0	0	0	NA	NA	NA	NA	NA	NA	NA	3
Papanagiotou et al. [36]	NA	NA	NA	0	0	8	2	3	0	10	8	2	0	0	NA	NA	NA	NA	10	0	0	NA
Singh et al. [37]	NA	1	13	10	2	0	2	1.214	0	14	14	0	0	0	9	2	1	2	11	0	3	NA
Hackl et al. [29]	6	2	11	13	0	0	0	NA	0	13	13	0	0	0	NA	NA	NA	NA	13	0	0	NA
Fuchs et al. [38]	1	1	5	NA	0	NA	NA	0.83	6	0	6	0	0	0	1	3	0	2	NA	NA	NA	NA
Janjua et al. [33]	NA	1	5	NA	0	NA	NA	NA	6	0	6	0	0	0	NA	NA	NA	NA	NA	NA	NA	NA
Bone graft alone																						
Hsu et al. [39]	NA	0	105	67	0	0	20	1	NA	NA	105	0	0	0	35	31	12	27	105	0	0	0
Li et al. [40]	NA	NA	NA	3	0	0	9	1.1	NA	NA	12	0	0	0	0	11	1	0	0	0	12	2
Marinelli et al. [41]	NA	6	51	42	0	15	0	1.3	NA	NA	0	57	0	0	7	12	12	26	57	0	0	7
Rollo et al. [42]	8	NA	NA	16	0	0	0	1	NA	NA	0	16	0	0	0	12	0	4	16	0	0	0
Pollon et al. [43]	6	3	13	NA	NA	NA	NA	2.25	NA	NA	16	0	0	0	0	15	0	1	16	0	0	16
Feng et al. [44]	NA	2	13	7	0	1	5	1.86	NA	NA	15	0	0	0	9	6	0	0	15	0	0	0
Takemoto et al. [35]	2	3	6	7	0	0	2	1.2	NA	NA	9	0	0	0	NA	NA	NA	NA	NA	NA	NA	1
Hackl et al. [29]	2	2	7	9	0	0	0	NA	NA	NA	9	0	0	0	NA	NA	NA	NA	9	0	0	NA
Fuchs et al. [38]	0	1	5	NA	0	NA	NA	1	NA	NA	6	0	0	0	0	3	0	3	NA	NA	NA	NA
